# The game-changing impact of *POLE* mutations in oncology—a review from a gynecologic oncology perspective

**DOI:** 10.3389/fonc.2024.1369189

**Published:** 2024-08-22

**Authors:** Johanna Kögl, Teresa L. Pan, Christian Marth, Alain G. Zeimet

**Affiliations:** Department of Obstetrics and Gynecology, Medical University of Innsbruck, Innsbruck, Austria

**Keywords:** POLE mutations, EDM, ultramutation, tumor mutational burden, hotspot mutations, endometrial cancer

## Abstract

Somatic mutations within the exonuclease proofreading domain (EDM) of the DNA polymerase Pol ϵ (*POLE*) gene are increasingly being discovered in ovarian, colorectal, urological, and, especially, endometrial carcinoma (EC), where these are found in up to 10% of the cases. In EC, there are five confirmed pathogenic somatic *POLE*-EDM mutations that are located at codons 286, 411, 297, 456, and 459, and these are called “hotspot” mutations. *POLE* mutant tumors are ultramutated entities with a frequency of base substitution mutations that is among the highest in human tumors. Interestingly, these mutations are associated with excellent clinical outcome in EC. An additional six “non-hotspot” *POLE*-EDM EC mutations are also considered pathogenic, and they also confer a favorable prognosis. Currently, de-escalation of adjuvant treatment is recommended for patients with EC with stage I–II tumors involving any of these 11 EDM mutations, even in patients with other clinicopathological risk factors. The high tumor mutational burden and the consequent increased infiltration of immune cells due to the overexpression of different neoantigens are probably responsible for the improved prognosis. Ongoing studies are examining *POLE* hotspot mutations among many non-gynecologic tumors, although the impact of such mutations on clinical outcomes is still a topic of debate. Therapeutic modalities for these hypermutated tumors are also an important consideration, including the need for or de-escalation of adjuvant treatments and the response to immune therapy. This review addresses the critical role of *POLE* mutations in gynecologic oncology and oncology in general, focusing on definitions, variants, underlying pathogenic mechanisms, upcoming developments in the field, and the clinic behavior associated with such mutations.

## 1 Introduction

The role of the DNA polymerase epsilon (POLE) in the correct replication of cellular DNA has been studied intensively (1). As a consequence of this research, the prognostic value of specific mutations in the *POLE* gene, so called “hotspot mutations,” has recently recognized, and this has revolutionized the management of endometrial and other cancers (2–4). A single missense mutation in a hotspot region of the gene can guide a clinician to reconsider the need for adjuvant therapy in cases, which, until recently, would have received treatments known to be associated with a high risk of complications (3). *POLE* mutant tumors are described in endometrial, ovarian, colorectal, and urological cancers (5–8). They lead to a ultramutated tumor phenotype and consistently demonstrate an excellent clinical outcome, especially in colorectal cancer (CRC) and endometrial cancer (EC) (5, 9).

**Figure 1:**
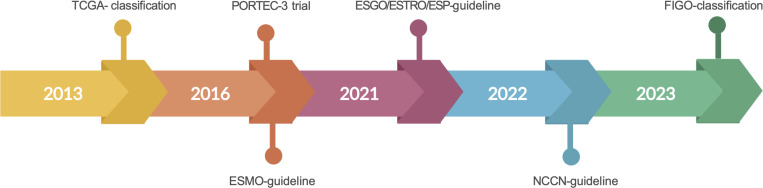
Major milestones in clinical practice in EC leading to substantial changes in the treatment of *POLE*-mutated ECs. TCGA, The Cancer Genome Atlas; ESMO, European Society for Medical Oncology; ESGO/ESTRO/ESP, European Society of Gynecological Oncology/European Society for Radiotherapy and Oncology/European Society of Pathology.

Historically, standard treatment of EC consisted of hysterectomy, bilateral salpingo-oophorectomy, and pelvic lymph node dissection followed by adjuvant therapy in the form of radiotherapy and/or chemotherapy based on final histology (10). However, management of EC has become more patient-specific over the past 10 years: differences in the histo-molecular classification predict prognosis and dictate whether adjuvant therapies are required or to be avoided (10) (**Figure 1**).

In 2013, The Cancer Genome Atlas (TCGA) identified a novel subgroup of ECs with unique mutations in *POLE* and associated unfavorable histomorphological features but, nevertheless, showing good survival outcomes. This new histo-molecular group was studied in the PORTEC-3 trial for patients with high-risk EC with Fédération International de Gynécologie et d’Obstétrique (FIGO) stage I–III, which investigated the benefit of adjuvant chemotherapy during and after radiotherapy over pelvic radiotherapy alone. The results of this trial showed that addition of adjuvant chemotherapy showed improved 5-year overall survival, especially in stage III patients. Crucially, molecular profiling revealed that *POLE*-mutated patients showed superior outcome, especially in stages I and II, irrespectively of their adjuvant treatment. This led to the author’s recommendation of de-escalation of adjuvant therapy for *POLE*-mutated patients (11, 12). These findings are limited by the relatively low number of *POLE*-mutated patients (12%) in general, and the fact that patients with stage III tumor disease showed superior outcome, if they had received a combined treatment. Furthermore, stage IV patients were not included in this trial. In 2016, the TCGA molecular classification was integrated into the ESMO (European Society for Medical Oncology) guideline, and this resulted in an updated risk classification for recurrence in stage I *POLE*-mutated ECs (13). The subgroup of *POLE*-mutated tumors was further integrated into the European Society of Gynecological Oncology (ESGO)/European Society for Radiotherapy and Oncology (ESTRO) and European Society of Pathology (ESP) guidelines for EC, resulting in specific changes in the recommendations for adjuvant treatment (3). The ESGO/ESTRO/ESP guidelines recommended that omission of adjuvant treatment should be considered for patients with stage I–II *POLE*-mutated EC. For the rare patients at stage III–IVA ECs with pathogenic *POLE* mutations, there are no reliable data on survival regarding omission of adjuvant treatment (3). In CRC as well, genetic testing for *POLE* was incorporated into the National Comprehensive Cancer Network guidelines in 2022 (14). Recently, this molecular classification has been integrated into the FIGO staging classification system of 2023. This shows the high clinical impact achieved by molecular characterization, including testing for *POLE* mutation, for optimal treatment of patients with EC. Although *POLE* mutant tumors tend to have a favorable outcome, there remain a number of questions to be answered: What is the physiological function and role of Pol ϵ? Is the good prognosis linked to the high mutational burden of *POLE*-mutated tumors? How is the correct annotation of the “*POLE* hotspot” mutations achieved? Is each *POLE* mutation pathogenic? What screening methods are available to determine *POLE* pathogenicity? What is the role of immunotherapy in *POLE*-mutated tumors and do *POLE* variants impact other tumor entities comparably? This review aims to discuss these fundamental questions and to highlight the current controversies related to this topic.

## 2 Molecular characteristics of POLE

Accurate replication of DNA prior to cell division is essential for maintaining genomic stability and for suppressing mutagenesis and tumor development (15). The high fidelity of eukaryotic DNA replication is due to a combination of highly accurate base incorporation and 3′-5′ exonuclease proofreading by the replicative DNA polymerases Pol δ and Pol ϵ and post-replication surveillance of the newly synthesized DNA by the mismatch repair (MMR) apparatus (5). In humans, Pol ϵ belongs to the B family polymerases, comprises four subunits, and is encoded by *POLE* (5). The proofreading function of Pol ϵ requires highly conserved motifs in their exonuclease domain (EDM), named exo-motifs, within which lie the catalytic site residues that are essential for exonuclease activity (5, 15). Misincorporation of a base into the leading strand leads to pausing of the polymerase ϵ and, consequently, to a switch from the catalytic site to the exonuclease domain, where the incorrect base is excised and replaced by the correct base (16).

Considering the close correlation between *POLE* mutations and increased mutation rates, it is important to define the tumor mutational burden (TMB) when describing tumor biology. TMB indicates the number of single mutations per megabase presented in the specimen. A high TMB describes a highly mutated tissue, which could be considered to have more aggressive biological behavior. More specifically, hypermutation is defined by a mutational load of 10 or more mutations per megabase (≥10 mut/Mb) (17), whereas ultramutated tumors show a frequency of base substitution mutations that is equal to or higher than 100 mutations per megabase (ultramutation ≥100 mut/Mb) (1).

## 3 Relevance of *POLE* mutations in endometrial cancer

### 3.1 Pathogenic *POLE*-EDM mutations in EC—definition, examples, and correlation with TMB

Somatic mutations within the *POLE* exonuclease proofreading domain (EDM) are found in 7%–12% of ECs (18), and these are always heterozygous changes (5, 15). Approximately 90% of the *POLE* proofreading mutations are in exons 9 and 13 and are recognized as pathogenic, i.e., driver mutations that are causal for tumor genesis by ultramutation (19). Generally, there are five common and confirmed pathogenic somatic *POLE-*EDM mutations that are located at codons **286**, **411**, **297**, **456**, and **459 (listed according to their decreasing prevalence)**. These are defined as **“hotspot” mutations**, but recurrent substitutions were also found in the complete TCGA EC cohort at codons 367, 424, 295, 368, 436, 444, 278, 428, 465, 352, 396, 402, 453, and 461 (decreasing frequency) (**Table 1**) (1). Most of these somatic substitutions lie within or close to the exo-motifs and will abolish exonuclease activity by causing perturbation of the DNA-binding pocket (8, 15). This affects protein function and subsequently increases the mutation rate (8, 15).

**Table 1:** Fifty-nine somatic *POLE–*exonuclease domain mutations (EDMs) in the TCGA cohort in EC with 11 pathogenic variants, listed by their frequency (1).

Nucleotide substitution	Frequency in the TCGA cohort*	Amino acid change	Mutation outcome†	Site	Exon
**c.857C>G**	21	**p.Pro286Arg**	Pathogen	**EDM** **hotspot**	9
**c.1231G>C**	13	**p.Val411Leu**	Pathogen	**EDM** **hotspot**	13
**c.890C>T**	3	**p.Ser297Phe**	Pathogen	**EDM** **hotspot**	9
**c.1366G>C**	2	**p.Ala456Pro**	Pathogen	**EDM** **hotspot**	14
**c.1376C>T**	2	**p.Ser459Phe**	Pathogen	**EDM** **hotspot**	14
**c.1100T>C**	2	p.Phe367Ser	Pathogen	EDM	11
**c.1270C>A**	2	p.Leu424Ile	Pathogen	EDM	13
**c.884T>G**	1	p.Met295Arg	Pathogen	EDM	9
**c.1102G>T**	1	p.Asp368Tyr	Pathogen	EDM	11
**c.1307C>G**	1	p.Pro436Arg	Pathogen	EDM	13
**c.1331T>A**	1	p.Met444Lys	Pathogen	EDM	13
**c.833C>T**	1	p.Thr278Met	Variant of unknown significance	EDM	9
**c.1270C>G**	1	p.Leu424Val	Variant of unknown significance	EDM	13
**c.1282G>A**	1	p.Ala428Thr	Variant of unknown significance	EDM	13
**c.1394C>T**	1	p.Ala465Val	Variant of unknown significance	EDM	14
**c.1056G>T**	1	p.Gln352His	Non-pathogenic	EDM	11
**c.1101dupT**	1	p.Asp368	Non-pathogenic	EDM	11
**c.1187A>G**	1	p.Glu396Gly	Non-pathogenic	EDM	12
**c.1204T>C**	1	p.Cys402Arg	Non-pathogenic	EDM	12
**c.1358A>G**	1	p.Gln453Arg	Non-pathogenic	EDM	13
**c.1382C>T**	1	p.Ser461Leu	Non-pathogenic	EDM	14

Pathogenic mutations are presented (n = 11); the most common of these are in bold (n = 5).

*TCGA (The Cancer Genome Atlas) cohort: 530 ECs, including 82 tumors with a somatic POLE mutation of which 59 were in the exonuclease domain (1).

†Pathogenicity was determined according to the developed scoring system by Leon-Castillo et al., in which characteristic features of the known pathogenic hotspot *POLE*-EDM (TMB > 100 mut/Mb, C>A ≥ 20%, T>G ≥ 4%, C>G ≤ 0.6%, and indels ≤ 5%) were taken into account. Tumors scored one point for each of the presented characteristic. A POLE-score ≥4 was considered as a pathogenic *POLE* mutation, a POLE-score = 3 as a variant of unknown significance and a score <3 as non-pathogenic *POLE* mutation.

*POLE* mutant ECs are by definition ultramutated and exhibit a frequency of base substitution mutations that is among the highest in human tumors (1). In general, although TMB in *POLE* mutant ECs is always elevated with a median value of 268 mut/Mb, overall TMB varies not only between different hotspot mutations but also among ECs with the same hotspot mutation (1). *POLE* mutant ECs present distinctive features such as a strong association with endometroid histology, high grade, microsatellite stability (MSS), a low proportion of small insertion and deletion mutations (indels), and a high proportion of C>A and T>G mutations in TCT and TTT tri-nucleotide contexts. These specific biological features are described as COSMIC signature 10 (15, 18, 20). ECs with *POLE* hotspot mutations are associated with a high prevalence of C>A, frequently exceeding 20%, and slightly lower T>G substitutions (13%) (1). Another unique aspect is the correlation with mutations in the Mismatch Repair Genes, which are commonly referred to as microsatellite (in)stability (MSI). Endometrial tumors with *POLE* mutations in one of the five most common codons and MSI have a high TMB (339 mut/Mb), whereas EC tumors with non-hotspot *POLE*-EDM mutations and MSI have a lower TMB (median, 207 mut/Mb) (1). As expected, ECs with mutations outside the EDM and concomitant MSI status display an even lower TMB of only 48.5 mut/Mb (1). This raises the question—is the better prognosis of *POLE* hotspot mutant ECs linked to the concomitant high TMB? High TMB causes genomic instability, which leads to an increased neoantigen expression and activation of the immune system (21). This is associated with a better immune response, which has also been seen in other solid tumors (22) and may explain at least, in part, the favorable clinical outcome of *POLE* mutant tumors (21). Incorrect annotation of a *POLE* variant can lead to erroneous classification of an endometrial carcinoma within the *POLE-*mutated subgroup, and this can impact the clinical management of the patient (2). How is correct annotation of *POLE* pathogenicity achieved and does pathogenicity of a hotspot and non-hotspot EDM mutation differ? Of note, the tumor cell content should be determined to provide most accurate information. Analysis of the TCGA endometrial carcinoma cohort using only ECs with a known pathogenic hotspot *POLE*-EDM as a “truth set” allowed the development of a scoring system, with well-defined cutoff points for examining pathogenicity of *POLE* variants (1, 2). In order to understand the scoring system, one has to understand that pathogenicity in this sense is causal for tumor ultramutation and, thus, favors a good clinical outcome. Taking into account the characteristic features of the known pathogenic hotspot *POLE*-EDM (TMB > 100 mut/Mb, C>A ≥ 20%, T>G ≥ 4%, C>G ≤ 0.6%, and indels ≤ 5%), a pragmatic scoring system was developed by Leon-Castillo et al., in which tumors scored 1 point for each of the presented characteristic (1). Hotspot *POLE* mutation scored 3–5 points, ECs with non-hotspot *POLE*-EDM mutations scored ≥3 points, whereas ECs with *POLE* mutations outside the exonuclease domain scored ≤2 points, due to the lack of genomic alterations (1). As pathogenicity increases with recurrent mutations, recurrence was also incorporated into the described POLE-score model. Based on this model, a *POLE* score ≥4 was used to define pathogenicity of *POLE* mutations in EC (1). ECs with a POLE-score ≤2 were classified as having non-pathogenic *POLE*-EDM, whereas ECs with a score of 3 were classified as variant of uncertain significance (1). Considering this POLE-score, only 11 of the 21 different *POLE* exonuclease domain variants in the TCGA cohort qualified as pathogenic (1, 2) (**Table 1**). This illustrates that the presence of a *POLE* mutation variant alone is not sufficient for classifying an endometrial carcinoma as *POLE-*mutated, let alone as pathogenic. The scoring system of Leon-Castillo has not been validated on independent and larger cohorts and, therefore, does not yet represent an international standardized tool for classifying the pathogenicity of hotspot versus non-hotspot EDM and non-EDM *POLE* mutations and for deciding on potentially de-escalating adjuvant treatment. So far, in the majority of the relatively small early retrospective studies, only the five hotspot mutations were generally classified as *POLE*mut and considered as a reference category in survival analysis. This was also the case for the data analysis of the larger PORTEC-3 Trial. However, the international meta-analysis on 294 *POLE*mut ECs included the 11 pathogenic *POLE*-EDM mutations described above and revealed a recurrence rate of 3.7%. However, one case was associated with both hotspot mutations P286R and V411L (23). The still recruiting phase II, RAINBO *POLE*mut-BLUE Trial (NCT05255653-4), also includes the 11 mentioned pathogenic EDM mutations. This first prospective trial of *POLE*mut EC is investigating complete omission of adjuvant therapy in lower-risk disease and de-escalation of treatment (observation versus radiotherapy, but not chemoradiation) in higher-risk disease (2). The outcome of RAINBO-BLUE will shed light on the mutations for which a de-escalation in the adjuvant treatment can be justified without concern. However, it is unclear if *POLE* pathogenicity is best assessed using a scoring system, TMB, or associated MSI status. Nevertheless, as whole-genome/exome sequencing techniques become more widely available, the current list of 11 pathogenic *POLE*-EDMs will increase in the near future, along with the need to precisely annotate defined *POLE* mutations. However, more evidence will be needed before new pathogenic mutations are included among those currently known to improve prognosis and thereby affect routine treatment decisions.

### 3.2 *POLE*mut EC in the context of the TCGA classification

Historically, EC has been classified into two subtypes (Bokhman classification) based on their clinical, endocrine, and histopathological characteristics (10, 24) In the last decade, molecular characteristics became components of Bokhman’s dualistic classification (25). However, the substantial heterogeneity of EC was not represented in this dichotomous classification (25). In 2013, analysis of TCGA identified four new genomic classes of ECs by combining information on somatic mutational burden and somatic copy number alterations (18). In recent years, Murali et al. (25) suggested that incorporation of molecular and genetic characteristics into the classification reflects tumor biology and prognostic outcome in EC more accurately. Traditionally, multiple factors such as histological subtype, G3 histology, myometrial invasion ≥50%, lymphovascular space invasion (LVSI), lymph node metastases, tumor diameter >2 cm, and presence of L1 cell adhesion molecule (L1CAM = CD171) have been identified as conferring high risk for recurrent disease (26). In more recent years, surrogate markers have been identified and incorporated in routine surgical pathology in order to allow identification of the four genomic classes of EC (2, 13). This entails sequencing of the exonuclease domain of *POLE* and assessment of the expression of MMR proteins (MLH1, PMS2, MSH2, and MSH6) and p53 by immunohistochemistry. This results in the current molecular classification of ECs into four new subgroups: *POLE*mut (ultramutated), MMR-deficient (MMRd), p53-abnormal (p53abn), and no specific molecular profile (NSMP), which represents the most heterogenous group (**Table 2**) (2, 13). The ProMisE (*Proactive Molecular Risk Classifier for Endometrial Cancer*) approach has been proven to be a reliable method for classifying tumors into the four subclassifications of TCGA. These four subgroups also show significant differences in clinical outcomes.

**Table 2:** International molecular classification of EC.

	*POLE*mut	MMRd	NSMP	p53aberrant
Prevalence in TCGA cohort	5%–15%	25%–30%	30%–40%	10%–25%
Associated histological features	High-grade endometrioid	Endometrioid miscellaneous, mostly low-grade	Low-grade endometrioid, clear cell	Serous, clear cell, high-grade endometrioid
Diagnostic test	Next-generation sequencing/Sanger/hotspot qPCR/hotspot: P286R, V411L, S297F, A456P, S459F	MMR-IHC: MLH1, MSH2, MSH6, PMS2; MSI assay: (qPCR) on DNA marker regions		p53-IHC
Associated molecular features	Ultramutated (≥100 mut/Mb)	Hypermutated (10–100 mut/Mb)	<10 mut/Mb	<10 mut/Mb
Associated clinical features	Low BMI Early stage Younger patients	High BMI 10% Lynch syndrome	High BMI	Low BMI Advanced stage Older patients
Prognosis	Excellent	Intermediate	Intermediate Grade-dependent	Poor

*POLE*mut, polymerase epsilon–ultramutated; MMRd, mismatch repair–deficient; NSMP, no specific molecular profile; TCGA, The Cancer Genome Atlas; IHC, immunohistochemistry; MMR, mismatch repair; MLH, MutL homolog; MSH, MutS protein homolog; PMS2, postmeiotic segregation increased 2; MSI, microsatellite Instability; BMI, body mass index; mut/Mb, mutations per megabases.

However, 3%–6% of EC tumors are referred to as multiple-classifiers, i.e., at first appearance, they belong to more than one molecular class and include those with combined *POLE*mut and p53abn, combined MMRd and p53abn, combined MMRd and *POLE*mut and a combination of all three defects (MMRd-*POLE*mut-p53abn) (27). Nevertheless, recently, a study has shown that multiple-classifier *POLE*mut-p53abn and MMRd-*POLE*mut can be categorized as single-classifier *POLE*mut and MMRd-p53abn as single-classifier MMRd EC (27). These findings result in a top-down classification hierarchy with *POLE*mut situated on top, followed by MMRd. In other words, if a patient’s tumor exhibits a p53abn status and a *POLE*mut, then no adjuvant therapy would be needed to treat such a patient in case of a stage I–II tumor. However, the current guidelines do not respect multiple classifiers yet. Furthermore, the data are yet limited on this subject.

The ESGO/ESTRO and the ESP 2021 guidelines integrated the molecular subgroups with traditional clinicopathological features into a novel risk stratification system for assessing the relative risk of recurrence and guiding treatment decisions (2, 3). The molecular characteristics have also been recently incorporated into the 2023 version of the FIGO staging classification system for EC. This new risk stratification system relies on the identification of surrogate markers that show a good relationship with clinical outcomes. However, implementation and interpretation of the surrogate markers in clinical practice remains challenging, especially for the *POLE* variants (2). The *POLE*-mutated class represents the smallest subgroup (7%) of ECs and is defined by somatic mutations in the catalytic subunit of the EDM of POLE (18, 28). Sixty percent of *POLE* ultramutated ECs are high-grade endometrioid lesions and 35% harbor a mutation of the *TP53* gene (10). Nevertheless, among *POLE* multiple-classifier cases, *POLE*mut outweighs the other described mutational defects. The new ESGO/ESTRO/ESP guidelines recommend that patients with stage I–II *POLE*-mutated EC do not need adjuvant treatment, irrespective of p53abn or MMR status. Such assessments are independent of traditional high risk factors (3). Patients with EC-classified *POLE*mut have an excellent prognosis and are expected to benefit from a de-escalation of postoperative adjuvant treatment, whereas patients with a *POLE*-unrelated p53abn EC have a worse prognosis and, thus, are expected to benefit from an intensification of treatment (4). The presence of a pathogenic *POLE* or *p53* mutation leads to a significant modification of the FIGO stage in early EC, in terms of downstaging or upstaging of disease (**Table 3**) (4).

**Table 3:** New 2023 FIGO endometrial cancer stage with molecular classification.

FIGO stage	Molecular findings in patients with early endometrial cancer (stages I and II after surgical staging)
**Stage IAm_POLEmut_**	*POLE*mut endometrial carcinoma, confined to the uterine corpus or with cervical extension, regardless of the degree of LVSI or histological type
**Stage IICm_p53abn_**	p53abn endometrial carcinoma confined to the uterine corpus with any myometrial invasion, with or without cervical invasion, and regardless of the degree of LVSI or histological type

*POLE*mut, polymerase epsilon–ultramutated; p53abn, p53abnormal; LVSI, lymphovascular space invasion.

Thus, stage II tumors with a *POLE*mut are now classified as Stage IAm_POLEmut_, whereas stage I tumors with a *p53* mutation are classified as Stage IICm_p53abn_ (**Table 3**).

## 4 Impact of *POLE* variants in other tumor entities and controversies

Given how the presence of a *POLE*-EDM mutation impacts the outcome and especially the therapeutic approach in EC, this raises the question—does this impact and approach generalize to other tumor entities? The oncological literature on this topic is expanding rapidly, and, almost in every field, there is an effort to identify possible pathogenic *POLE*-mutated variants.

### 4.1 Ovarian cancer

There is an increasing interest in *POLE* mutations in ovarian cancer (OC). Four of the five *POLE* hotspot mutations (P286R, S297F, V411L, and A456P) have been found in an OC cohort of 195 patients, with *POLE* mutations found in 1.5% of tumors. All such tumors were of the endometroid histotype and had an earlier onset with an average age at diagnosis of 48 years (6). However, Parra-Herran et al. (29) analyzed *POLE*-EDM mutations in ovarian clear cell cancer (OCCC) but could not detect any pathogenic *POLE* mutation in a total of 47 cases. Nevertheless, several variants of unknown significance were detected. Among endometroid OCs, the overall incidence of *POLE* mutations appears to be up to 8% with a high proportion of heterozygous *POLE* p.297 mutations (30). Furthermore, in endometroid OCs, *POLE*-mutated cases present at an early stage (75% at FIGO stage I), and none was staged FIGO III or higher. All mutations were somatic mutations at P286R and V411L, and the patients had an uneventful clinical course without recurrence. These data are extrapolated from three single center studies and are not inadequate for establishing the overall prevalence of *POLE* mutations among all OCs. A study by Leskela et al. (31) determined MMR, p53 and *POLE-*EDM in early stage endometrioid OCs based on the molecular classification used for EC. Five tumors (3%) were double classifiers, whereas most of the cohort (66%) belonged to the NSMP (no specific molecular profile) group. In the *POLE-*EDM–mutated group, tumors (overall 8%) were ultramutated and showed higher infiltrations of CD8-lymphocytes compared with the rest of the cohort. Although the prognosis did not differ among subgroups in the multivariate analysis, a tendency toward better prognosis in *POLE*-mutated and a worse prognosis in p53 abnormal tumors was noted (31).

#### 4.1.1 Conclusion—*POLE* in OC

The role of *POLE*-EDM mutations in endometroid OC is currently emerging in the oncological literature. It has been shown that *POLE* mutations are more common in endometroid OC and are associated with younger age and earlier stage at diagnosis. Whether *POLE* mutations also lead to a better prognosis in endometroid OCs remains unconfirmed, and there appears to be a rationale for testing patients with early onset endometroid OCs, as they could be candidates for immunotherapy.

### 4.2 Colorectal cancer and urological cancers (prostate and bladder cancers)

The role of *POLE*-EDM mutations with regard to pathogenesis, prognosis, and therapeutic options has been widely investigated in CRC over the past few years (9). Ultramutated phenotypes with a high TMB (cutoff >150mut/Mb) can help to identify possible *POLE*-mutated CRCs and to guide selected screening. Furthermore, CRC *POLE*-mutated tumors are mainly diagnosed at relatively younger age (before 55 years) and at an early stage (14, 32–34).

Genomic studies of urothelial bladder carcinomas from the TCGA cohort have revealed a prevalence of 6.1% for *POLE* mutations (7). These *POLE* mutant urological cancers present known pathogenic hotspot mutations with a high TMB and a durable response to ICI (immune checkpoint inhibitor) therapy (35, 36).

A summary of the known *POLE*-EDM mutations in different tumor entities is presented in **Table 4**.

**Table 4:** Described pathogenic *POLE-*EDM mutations in different tumor entities.

Tumor location	Described mutation¹	Clinical characteristics	Prevalence	Level of evidence
Endometroid ovarian cancer	P286R, S297F, V411L, A456P No cases in OCCC	Younger age at diagnosis; early stage; lower recurrence rates; higher CD8-lympocytes infiltration	1.5%–8%	Retrospective single center studies (6, 29–31)
Colorectal cancer	P286R, V411L, S459F	Younger age at diagnosis; early stage	About 6%	Meta-analysis from retrospective single center studies (9, 14, 32–34)
Urothelial bladder carcinomas	P286R	Durable response to ICI therapy	About 6%	Retrospective single center studies (7, 35, 36)

¹From the five defined pathogenic “hotspot” mutations; OCCC, ovarian clear cell cancer; ICI, immune checkpoint inhibitor.

## 5 The problem with screening techniques for identifying *POLE*-EDM variants and new possible surrogate markers (TILs and Immunoscores)

Evaluation of a pathogenic *POLE* mutation remains challenging, as parameters and methods that allow a standard procedure in clinical practice have not been validated as yet. Whole-exome or whole-genome sequencing (WES/WGS) by Sanger or next-generation-sequencing can be used to identify *POLE* mutations in the exonuclease domain (exons 9–14). However, these methods are time-consuming, not widely available, and expensive; require expertise; and, therefore, limit routine use in current clinical practice. Estimation of pathogenicity of somatic *POLE* mutations in the absence of exome and genome sequencing has been carried out by some authors by using *in silico* prediction tools (1). Although this seems to be a feasible technique, the setting prognosis relies on the sequencing tools that have been used. Clinical practice requires a *POLE* testing method that is not only affordable, with a fast turnaround time, but also easy to interpret and implement. Therefore, sequencing methods restricted to the analysis of the hotspot *POLE* exonuclease domain mutations could present an alternative technique and have been developed recently by several research groups. Deveraux et al. (37, 38), for example, use a single-gene *POLE* hotspot SNaPshot assay in their routine prospective molecular classification of ECs. This technique involves an initial PCR amplification of the relevant gene target regions of the *POLE*-EDMs, followed by multiplexed single-nucleotide primer extension (38). Van den Heerik et al. (39) created a quantitative polymerase chain reaction (qPCR) assay for pathogenic *POLE* mutations (*QPOLE*). So far, there is no standardized method that allows determination of *POLE* mutations in ECs in clinical practice. Moreover, a workflow that reports both the molecular and histologic findings in an integrative manner is still not available. However, the integration of molecular classification together with clinicopathologic features into the ESGO/ESTRO/ESP guidelines and into the novel 2023 FIGO staging classification system shows the high clinical impact that testing of *POLE* mutation has in the patients’ treatment and management. This issue cannot be ignored any longer. It is critically necessary that the clinical assay used in daily practice reliably identifies *POLE* mutations in the hotspot *POLE*-EDM as their role in tumor biology and their therapeutical consequences are known. However, as new hotspot *POLE*-EDM mutations continue to emerge, their clinical role must be rapidly evaluated, and they should be incorporated into validated assays as appropriate. One indirect approach to identify *POLE*-mutated cancers is by looking at the number of tumor-infiltrating lymphocytes (TILs), as we know that a highly mutated microenvironment expresses more antigens and, therefore, activates the host’s immune system. For example, in CRC cases, MSI tumors were TIL-high (≥4 lymphocytes per high-power field), in 68% of cases with a TMB of 54 mut/Mb, whereas MSS CRCs were only TIL-high in 4.5% of cases. In contrast, MSS CRC tumors with *POLE/POLD1* pathogenic variants were TIL-high in 82% of cases and had a TMB over 150 mut/Mb. These differences in tumoral immunity provide the rationale for immunotherapy (40). A possible way to screen for patients who might benefit from immunotherapy even if they are MMR-intact could be by using an immune microenvironment evaluation system such as that described by Galon et al. (41). In CRC, the Immunoscore (IS) has been shown to be a prognostic factor superior to the previous tumor, node, metastasis (TNM) classification of malignant tumors. There are ongoing validation and promotion initiatives to increase the use of IS in routine clinical settings (41). There are several ongoing clinical trials assessing the efficacy of ICI therapy for treating patients with *POLE/POLD1* mutations, especially for metastatic CRC (40, 42–44). Different systems of immunoscoring have also found application in gastric and endometrial cancer (45), but further prognostic studies are needed to validate the routine use of ISs in routine clinical practice.

## 6 Effects of *POLE* mutations on prognosis and their therapeutic consequences—role of immunotherapy

In EC, the ultramutated phenotype caused by *POLE*-EDM mutations has been shown to cause a “self-limiting” tumor progression with excellent prognosis after surgery. This has been demonstrated even without adjuvant treatment in patients previously classified as “high” or “intermediate-high” risk (10, 46). The Identification of pathogenic *POLE* mutations in early-stage EC also plays a crucial role when considering fertility sparing treatments (FSTs) such as hormonal therapy or hysteroscopic resection in young women (47, 48). Improving risk stratification for FST is one of the future targets, because the molecular classification and different molecular markers emerging are changing the risk profile assessment for patients. A preliminary molecular analysis of an endometrial biopsy is, therefore, necessary in patients with desire to conceive or in case of organ sparing. Pathologists should systematically perform a molecular analysis of hysteroscopic biopsy samples, including the sequencing for the presence of the *POLE* mutation. In OC of the endometrioid histological subtype, there is reason to believe that *POLE* mutations lead to a better prognosis, but further confirmation is needed (49). Although there are many molecular studies on *POLE*-mutated CRCs, data on the clinical implications of the *POLE* ultramutated phenotype are lacking. In some studies of *POLE*-EDM–mutated stage II CRCs, a robust intratumoral T-cell response was detected in a small subset of cases with excellent outcomes (50). However, a significantly better prognosis across all stages of CRC has not been confirmed as yet (as in the case of ECs). Therefore, the therapeutic management of CRC is currently not impacted by the presence of a *POLE* mutation. In other solid tumors such as pancreatic cancer, mutations in the hotspot regions of *POLE* are very rare events. In advanced pancreatic cancer, it is highly unlikely that *POLE* mutations contribute to genetic instability; therefore, *POLE* mutations do not serve as a relevant biomarker and should not be tested for on a regular basis (51). In a totally different oncologic entity, namely, high-grade gliomas (HGGs), a subgroup based on somatic *POLE* mutations has been identified. Such cases are genomically, histologically, and clinically different from the other HGGs and exhibit an improved prognosis (52). Most recently, two trials have clarified the role of ICI for the treatment of EC. These two big randomized phase III trials (RUBY and GY018) with still limited follow-up did so far not change the practice for *POLE*-mutated EC (53). Over the last several years, an enormous effort has been made with regard to EC to find new biomarkers that can accurately identify patients who can benefit from immunotherapy even if they are not MMR deficient. *In silico* analysis has proved that *POLE* mutant cancers display more antigenic neoepitopes than other ECs, providing a potential rational for POLE immunogenicity (54). Yet *POLE*-mutated tumors are currently not a recommended indication for ICIs even in advanced and metastatic EC cases. On the other hand, CRCs have recently received approval for treatment of MSI CRC with ICIs. Assessment of POLE status may help guide therapeutic decisions for tumors with high TMB and intact MMR. Recent reports have shown that, even in advanced CRC or multiresistent disease, patients can significantly benefit from ICIs, if they harbor a pathogenic *POLE* mutation (55–57). A case report of a high-grade CRC with *POLE*-EDM (P286R) mutation and TMB of 119 mut/Mb described triple-chemotherapy being ineffective, whereas ICIs had a significant impact on progression-free survival (PFS) (55). Another example involved the case of a 24-year-old male patient with an aggressive stage IV high-grade, poorly differentiated CRC, where response was complete and durable (over 48 months) with a single-agent ICI after rapidly progression with standard chemotherapy. Genetic testing of this case revealed a P286R *POLE* mutation and an elevated TMB of 126 mut/Mb (57). These cases highlight the interplay between genetic instability and immune-checkpoint blockade. In a case report of OC (OCCC inoperable at stage IIIB), resistant to platinum-based chemotherapy, the same P286R *POLE* mutation was found, and the third-line treatment attempt with a programmed cell death protein 1 (PD-1) inhibitor showed a tumor with postoperative pathologic complete response. The patient achieved a PFS of 29 months under maintenance with ICI therapy (58). Therefore, even if the ProMisE classification does not find an application in OC, the simple presence of *POLE* hotspot mutation can, in exceptional cases, guide for treatment with ICIs.

## 7 Ongoing trials for *POLE*-mutated endometrial cancers

At the present, it is important to acknowledge that the changes in clinical treatment of *POLE*-mutated ECs as recommended by the ESGO/ESTRO/ESP guideline of 2021 are based on much less data than what is typically used for such profound and nearly dogmatic shifts in clinical care. Evaluation of the benefit of these clinical and therapeutic changes through prospective studies can help provide more information on this issue. Currently, prospective clinical trials, such as PORTEC-4 and TAPER, are ongoing and will shed light and yield more insights whether *POLE*-mutated ECs have a favorable outcome even without or de-escalated adjuvant treatment. The TAPER trial is an interventional study based on tailored adjuvant therapy in *POLE*-mutated and *p53* wild-type/NSMP early-stage EC. Its primary objective is to determine if women with cancers with the specific molecular characteristics who underwent adequate surgery have a relative low risk (lower than 5%) of pelvic and vaginal recurrence at 3 years with no or de-escalated adjuvant treatment (59). The PORTEC-4a trial is a randomized phase III trial of molecular profile-based versus standard recommendations for adjuvant radiotherapy in stage I EC and is focused on cancers classified as high-intermediate risk according to the ESMO-ESGO-ESTRO consensus of 2015. The primary endpoint is vaginal recurrence, and other oncologic but secondary endpoints are recurrence-free and overall survival, as well as pelvic and distant recurrence. As, in this study, all patients with *POLE*-mutated cancers are allocated without restrictions to the “favorable molecular risk group” with omission of vaginal brachytherapy and external pelvic beam radiotherapy, this trial is very likely to extend our knowledge on prognosis of *POLE*-mutated cancers at least in this prespecified subset of patients. First results are expected at the end of 2024 or in the spring 2025, and study completion is set for 2028 with 550 patients enrolled (60). Lastly, the blue arm of the RAINBO trial is also focused at *POLE*-mutated ECs. The RAINBO program is a platform of four international clinical trials and an overarching research program, including a randomized PHASE III trial with three arms for p53-abn EC (red), MMRd EC (green), and NSMP (orange) ECs. The *POLE*mut-BLUE trial is a phase II trial in which the safety of de-escalation of adjuvant therapy is investigated for women with stage I–III *POLE*mut EC. This trial will evaluate no adjuvant therapy for lower-risk disease and no adjuvant therapy or radiotherapy alone with omission of concomitant chemotherapy for higher-risk disease. The primary endpoint of this trial will be pelvic recurrence at 3 years. This study is in the recruiting phase and main trial results are expected in 2028 (61).

A summary of relevant concluded or ongoing trials about ICIs and adjuvant treatments in EC based on molecular classification is shown in **Table 5**.

**Table 5:** A summary of relevant concluded or ongoing trials about ICIs and adjuvant treatments in EC based on molecular classification.

Trial	Full name of trial	Results
PORTEC-4a	Molecular profile-based versus standard adjuvant radiotherapy in EC	Study completion estimated: 31 December 2028
RAINBO	Refining adjuvant treatment in EC based on molecular features	Study completion estimated: 1 January 2023
TAPER	Adjuvant therapy in *POLE*-mutated and p53 wild-type/NSMP early stage EC	Study completion estimated: 30 June 2029
RUBY	A study to evaluate dostarlimab plus carboplatin-paclitaxel versus placebo plus carboplatin-paclitaxel in participants with recurrent or primary advanced EC (RUBY)	Conclusion: Dostarlimab plus carboplatin-paclitaxel significantly increased PFS among patients with primary advanced or recurrent EC, with a substantial benefit in the dMMR–MSI-H population.
GY018	Testing the addition of the immunotherapy drug pembrolizumab to the usual chemotherapy treatment (paclitaxel and carboplatin) in stage III–IV or recurrent EC	Conclusion: In patients with advanced or recurrent EC, the addition of pembrolizumab to standard chemotherapy resulted in significantly longer PFS than with chemotherapy alone.

## 8 Conclusion

*POLE* mutational status in EC is of great clinical interest. It determines the prognosis of the patient, and the FIGO classification system 2023 stipulates that its presence should result in a significant de-escalation of adjuvant treatment. In the future, unresolved questions will be better answered by the results of the *POLE*mut-BLUE arm of the prospective phase II RAINBO trial, where even stage III patients are included. *POLE*-mut ECs are assigned depending on risk status either to an observational arm with complete omission of adjuvant treatment or to radiotherapy alone. We describe in detail that the sole presence of a *POLE* variant is not sufficient to classify a tumor as *POLE*mutated or to classify a *POLE* mutation as pathogenic. For that, the exact localization of the mutation in the *POLE* gene needs to be known. It has a higher probability of being pathogenic when it is located in the EDM of Pol ϵ. However, unanswered questions, such as the exact molecular pathways associated with the good prognosis of *POLE* mutations, remain unclear and, therefore, unmentioned. Due to inconsistencies in *POLE* mutation testing and its interpretation, the study investigators have advocated a concomitant TMB determination in order to underscore the pathogenicity of the *POLE*-EDM mutation. Performed as a routine procedure, this appears to be one of the best and easiest approaches to identify new pathogenic *POLE* mutations. We are hopeful that the increasing knowledge on the exact oncologic driver qualities of *POLE* mutations together with the outcome of mentioned prospective clinical research will enable reassured avoidance or at least de-escalation of adjuvant treatment in EC without harming patients by under- or overtreatment.

## References

[1] León-Castillo A Britton H McConechy MK McAlpine JN Nout R Kommoss S. Interpretation of somatic POLE mutations in endometrial carcinoma. J Pathol. (2020) 250:323–35. doi: 10.1002/path.5372 PMC7065171 31829442

[2] Léon-Castillo A. Update in the molecular classification of endometrial carcinoma. Int J Gynecol Cancer. (2023) 33:333–42. doi: 10.1136/ijgc-2022-003772 36878561

[3] Concin N Matias-Guiu X Vergote I Cibula D Mirza MR Marnitz S. ESGO/ESTRO/ESP guidelines for the management of patients with endometrial carcinoma. Int J Gynecol Cancer. (2021) 31:12–39. doi: 10.1136/ijgc-2020-002230 33397713

[4] Berek JS Matias-Guiu X Creutzberg C Fotopoulou C Gaffney D Kehoe S. FIGO staging of endometrial cancer: 2023. Int J Gynecol Obs. (2023) 162:383–94. doi: 10.1002/ijgo.14923 37337978

[5] Rayner E Van Gool IC Palles C Kearsey SE Bosse T Tomlinson I. A panoply of errors: Polymerase proofreading domain mutations in cancer. Nat Rev Cancer. (2016) 16:71–81. doi: 10.1038/nrc.2015.12 26822575

[6] Davila JI Chanana P Sarangi V Fogarty ZC Weroha SJ Guo R. Frequent POLE-driven hypermutation in ovarian endometrioid cancer revealed by mutational signatures in RNA sequencing. BMC Med Genomics. (2021) 14:165. doi: 10.1186/s12920-021-01017-7 34158040 PMC8218518

[7] Voutsadakis IA. Urothelial bladder carcinomas with high tumor mutation burden have a better prognosis and targetable molecular defects beyond immunotherapies. Curr Oncol. (2022) 29:1390–407. doi: 10.3390/curroncol29030117 PMC8947463 35323317

[8] Shinbrot E Henninger EE Weinhold N Covington KR Göksenin AY Schultz N. Exonuclease mutations in DNA polymerase epsilon reveal replication strand specific mutation patterns and human origins of replication. Genome Res. (2014) 24:1740–50. doi: 10.1101/gr.174789.114 PMC4216916 25228659

[9] Domingo E Freeman-Mills L Rayner E Glaire M Briggs S Vermeulen L. Somatic POLE proofreading domain mutation, immune response, and prognosis in colorectal cancer: a retrospective, pooled biomarker study. Lancet Gastroenterol Hepatol. (2016) 1:207–16. doi: 10.1016/S2468-1253(16)30014-0 28404093

[10] Morice P Leary A Creutzberg C Abu-Rustum N Darai E. Endometrial cancer. Lancet. (2016) 387:1094–108. doi: 10.1016/S0140-6736(15)00130-0 26354523

[11] Leon-Castillo A De Boer SM Powell ME Mileshkin LR Mackay HJ Leary A. Molecular classification of the PORTEC-3 trial for high-risk endometrial cancer: Impact on prognosis and benefit from adjuvant therapy. J Clin Oncol. (2020) 38:3388–97. doi: 10.1200/JCO.20.00549 PMC7527156 32749941

[12] de Boer SM Powell ME Mileshkin L Katsaros D Bessette P Haie-Meder C. Adjuvant chemoradiotherapy versus radiotherapy alone in women with high-risk endometrial cancer (PORTEC-3): patterns of recurrence and *post-hoc* survival analysis of a randomised phase 3 trial. Lancet Oncol. (2019) 20:1273–85. doi: 10.1016/S1470-2045(19)30395-X PMC6722042 31345626

[13] Oaknin A Bosse TJ Creutzberg CL Giornelli G Harter P Joly F. Endometrial cancer: ESMO Clinical Practice Guideline for diagnosis, treatment and follow-up ☆. Ann Oncol. (2022) 33:860–77. doi: 10.1016/j.annonc.2022.05.009 35690222

[14] Weiss JM Gupta S Burke CA Axell L Chen LM Chung DC. NCCN guidelines^®^ Insights: genetic/familial high-risk assessment: colorectal, version 1.2021. J Natl Compr Canc Netw. (2021) 19:1122–32. doi: 10.1164/jnccn.2021.0048 34666312

[15] Church DN Briggs SEW Palles C Domingo E Kearsey SJ Grimes JM. DNA polymerase ϵ and δ exonuclease domain mutations in endometrial cancer. Hum Mol Genet. (2013) 22:2820–8. doi: 10.1093/hmg/ddt131 PMC3690967 23528559

[16] Ganai RA Bylund GO Johansson E. Switching between polymerase and exonuclease sites in DNA polymerase ϵ. Nucleic Acids Res. (2015) 43:932–42. doi: 10.1093/nar/gku1353 PMC4333401 25550436

[17] Campbell BB Light N Fabrizio D Zatzman M Fuligni F de Borja R. Comprehensive analysis of hypermutation in human cancer. Cell. (2017) 171:1042–1056.e10. doi: 10.1016/j.cell.2017.09.048 29056344 PMC5849393

[18] Getz G Gabriel SB Cibulskis K Lander E Sivachenko A Sougnez C. Integrated genomic characterization of endometrial carcinoma. Nature. (2013) 497:67–73. doi: 10.1038/nature12113 23636398 PMC3704730

[19] Church DN Stelloo E Nout RA Valtcheva N Depreeuw J Ter Haar N. Prognostic significance of POLE proofreading mutations in endometrial cancer. J Natl Cancer Inst. (2015) 107:402. doi: 10.1093/jnci/dju402 25505230 PMC4301706

[20] Fang H Barbour JA Poulos RC Katainen R Aaltonen LA Wong JWH. Mutational processes of distinct POLE exonuclease domain mutants drive an enrichment of a specific TP53 mutation in colorectal cancer. PloS Genet. (2020) 16:e1008572. doi: 10.1371/journal.pgen.1008572 32012149 PMC7018097

[21] Imboden S Nastic D Ghaderi M Rydberg F Rau TT Mueller MD. Phenotype of POLE-mutated endometrial cancer. PloS One. (2019) 14:e0214318. doi: 10.1371/journal.pone.0214318 30917185 PMC6436745

[22] Strickler JH Hanks BA Khasraw M. Tumor mutational burden as a predictor of immunotherapy response: is more always better? Clin Cancer Res. (2021) 27:1236–41. doi: 10.1158/1078-0432.CCR-20-3054 PMC9912042 33199494

[23] McAlpine JN Chiu DS Nout RA Church DN Schmidt P Lam S. Evaluation of treatment effects in patients with endometrial cancer and POLE mutations: An individual patient data meta-analysis. Cancer. (2021) 127:2409–22. doi: 10.1002/cncr.33516 33793971

[24] Bokhman JV. Two pathogenetic types of endometrial carcinoma. Gynecol Oncol. (1983) 15:10–7. doi: 10.1016/0090-8258(83)90111-7 6822361

[25] Murali R Soslow RA Weigelt B. Classification of endometrial carcinoma: more than two types. Lancet Oncol. (2014) 15:e268–78. doi: 10.1016/S1470-2045(13)70591-6 24872110

[26] Zeimet AG Reimer D Huszar M Winterhoff B Puistola U Azim SA. L1CAM in early-stage type i endometrial cancer: Results of a large multicenter evaluation. J Natl Cancer Inst. (2013) 105:1142–50. doi: 10.1093/jnci/djt144 23781004

[27] León-Castillo A Gilvazquez E Nout R Smit VT McAlpine JN McConechy M. Clinicopathological and molecular characterisation of ‘multiple-classifier’ endometrial carcinomas. J Pathol. (2020) 250:312–22. doi: 10.1002/path.5373 PMC7065184 31829447

[28] Hussein YR Weigelt B Levine DA Schoolmeester JK Dao LN Balzer BL. Clinicopathological analysis of endometrial carcinomas harboring somatic POLE exonuclease domain mutations. Mod Pathol. (2015) 28:505–14. doi: 10.1038/modpathol.2014.143 25394778

[29] Parra-Herran C Bassiouny D Lerner-Ellis J Olkhov-Mitsel E Ismiil N Hogen L. P53, mismatch repair protein, and POLE abnormalities in ovarian clear cell carcinoma: an outcome-based clinicopathologic analysis. Am J Surg Pathol. (2019) 43:1591–9. doi: 10.1097/PAS.0000000000001328 31335355

[30] Zou Y Liu FY Liu H Wang F Li W Huang MZ. Frequent POLE1 p.S297F mutation in Chinese patients with ovarian endometrioid carcinoma. Mutat Res Mol Mech Mutagen. (2014) 761:49–52. doi: 10.1016/j.mrfmmm.2014.01.003 24472300

[31] Leskela S Romero I Rosa-Rosa JM Caniego-Casas T Cristobal E Pérez-Mies B. Molecular heterogeneity of endometrioid ovarian carcinoma. Am J Surg Pathol. (2020) 44:982–90. doi: 10.1097/PAS.0000000000001478 32384322

[32] Stadler ZK Battaglin F Middha S Hechtman JF Tran C Cercek A. Reliable detection of mismatch repair deficiency in colorectal cancers using mutational load in next-generation sequencing panels. J Clin Oncol. (2016) 34:2141–7. doi: 10.1200/JCO.2015.65.1067 PMC4962706 27022117

[33] Kawai T Nyuya A Mori Y Tanaka T Tanioka H Yasui K. Clinical and epigenetic features of colorectal cancer patients with somatic POLE proofreading mutations. Clin Epigenetics. (2021) 13:117. doi: 10.1186/s13148-021-01104-7 34034807 PMC8146650

[34] Hu H Cai W Wu D Hu W Wang L. Ultra-mutated colorectal cancer patients with POLE driver mutations exhibit distinct clinical patterns. Cancer Med. (2021) 10:135–42. doi: 10.1002/cam4.3579 PMC7826451 33125191

[35] Lee L Ali S Genega E Reed D Sokol E Mathew P. Aggressive-variant microsatellite-stable POLE mutant prostate cancer with high mutation burden and durable response to immune checkpoint inhibitor therapy. JCO Precis Oncol. (2018) 2:1–8. doi: 10.1200/PO.17.00097 35135110

[36] Halbert B Einstein DJ. Hot or not: tumor mutational burden (TMB) as a biomarker of immunotherapy response in genitourinary cancers. Urology. (2021) 147:119–26. doi: 10.1016/j.urology.2020.10.030 33137348

[37] Devereaux KA Steiner DF Ho C Gomez AJ Gilks B Longacre TA. A multiplex SNaPshot assay is a rapid and cost-effective method for detecting POLE exonuclease domain mutations in endometrial carcinoma. Int J Gynecol Pathol. (2022) 41:541–51. doi: 10.1097/PGP.0000000000000841 34907997

[38] Devereaux KA Weiel JJ Pors J Steiner DF Ho C Charu V. Prospective molecular classification of endometrial carcinomas: institutional implementation, practice, and clinical experience. Mod Pathol. (2022) 35:688–96. doi: 10.1038/s41379-021-00963-y PMC12817303 34743187

[39] Van den Heerik ASVM Ter Haar NT Vermij L Jobsen JJ Brinkhuis M Roothaan SM. QPOLE : A quick, simple, and cheap alternative for POLE sequencing in endometrial cancer by multiplex genotyping quantitative polymerase chain reaction. JCO Glob Oncol. (2023) 9:1–11. doi: 10.1200/GO.22.00384 PMC10497260 37229628

[40] Keshinro A Vanderbilt C Kim JK Firat C Chen CT Yaeger R. Tumor-infiltrating lymphocytes, tumor mutational burden, and genetic alterations in microsatellite unstable, microsatellite stable, or mutant POLE/POLD1 colon cancer. JCO Precis Oncol. (2021) 5:817–26. doi: 10.1200/PO.20.00456 PMC8232557 34250404

[41] Galon J Mlecnik B Bindea G Angell HK Berger A Lagorce C. Towards the introduction of the ‘Immunoscore’ in the classification of Malignant tumours. J Pathol. (2014) 232:199–209. doi: 10.1002/path.4287 24122236 PMC4255306

[42] Forgó E Gomez AJ Steiner D Zehnder J Longacre TA. Morphological, immunophenotypical and molecular features of hypermutation in colorectal carcinomas with mutations in DNA polymerase ϵ (POLE). Histopathology. (2020) 76:366–74. doi: 10.1111/his.13984 31479159

[43] Kim JH Kim SY Baek JY Cha YJ Ahn JB Kim HS. A phase II study of avelumab monotherapy in patients with mismatch repair-deficient/microsatellite instability-high or POLE-mutated metastatic or unresectable colorectal cancer. Cancer Res Treat. (2020) 52:1135–44. doi: 10.4143/crt.2020.218 PMC7577804 32340084

[44] Oh CR Kim JE Hong YS Kim SY Ahn JB Baek JY. Phase II study of durvalumab monotherapy in patients with previously treated microsatellite instability-high/mismatch repair-deficient or POLE-mutated metastatic or unresectable colorectal cancer. Int J Cancer. (2022) 150:2038–45. doi: 10.1002/ijc.33966 35179785

[45] Jiang Y Zhang Q Hu Y Li T Yu J Zhao L. ImmunoScore signature. Ann Surg. (2018) 267:504–13. doi: 10.1097/SLA.0000000000002116 28002059

[46] Meng B Hoang LN Mcintyre JB Duggan MA Nelson GS Lee CH. POLE exonuclease domain mutation predicts long progression-free survival in grade 3 endometrioid carcinoma of the endometrium. Gynecol Oncol. (2014) 134:15–9. doi: 10.1016/j.ygyno.2014.05.006 24844595

[47] Ronsini C Mosca L Iavarone I Nicoletti R Vinci D Carotenuto RM. Oncological outcomes in fertility-sparing treatment in stage IA-G2 endometrial cancer. Front Oncol. (2022) 12:965029. doi: 10.3389/fonc.2022.965029 36185260 PMC9524219

[48] Cavaliere AF Perelli F Zaami S D’Indinosante M Turrini I Giusti M. Fertility sparing treatments in endometrial cancer patients: The potential role of the new molecular classification. Int J Mol Sci. (2021) 22:12248. doi: 10.3390/ijms222212248 34830129 PMC8625356

[49] Hoang LN McConechy MK Köbel M Anglesio M Senz J Maassen M. Polymerase epsilon exonuclease domain mutations in ovarian endometrioid carcinoma. Int J Gynecol Cancer. (2015) 25:1187–93. doi: 10.1097/IGC.0000000000000492 26166557

[50] Mo S Ma X Li Y Zhang L Hou T Han-Zhang H. Somatic POLE exonuclease domain mutations elicit enhanced intratumoral immune responses in stage II colorectal cancer. J Immunother Cancer. (2020) 8:881. doi: 10.1136/jitc-2020-000881 PMC7454238 32859741

[51] Guenther M Veninga V Kumbrink J Haas M Westphalen CB Kruger S. POLE gene hotspot mutations in advanced pancreatic cancer. J Cancer Res Clin Oncol. (2018) 144:2161–6. doi: 10.1007/s00432-018-2746-x PMC11813388 30194485

[52] Erson-Omay EZ Cąglayan AO Schultz N Weinhold N Omay SB Özduman K. Somatic POLE mutations cause an ultramutated giant cell high-grade glioma subtype with better prognosis. Neuro Oncol. (2015) 17:1356–64. doi: 10.1093/neuonc/nov027 PMC4578578 25740784

[53] Eskander RN Sill MW Beffa L Moore RG Hope JM Musa FB. Pembrolizumab plus chemotherapy in advanced endometrial cancer. N Engl J Med. (2023) 388:2159–70. doi: 10.1056/NEJMoa2302312 PMC10351614 36972022

[54] Van Gool IC Eggink FA Freeman-Mills L Stelloo E Marchi E De Bruyn M. POLE proofreading mutations elicit an antitumor immune response in endometrial cancer. Clin Cancer Res. (2015) 21:3347–55. doi: 10.1158/1078-0432.CCR-15-0057 PMC4627582 25878334

[55] Xiang D Fu G Chen Y Chu X. Case report: POLE (P286R) mutation in a case of recurrent intestinal leakage and its treatment. Front Oncol. (2023) 13:1028179. doi: 10.3389/fonc.2023.1028179 37007102 PMC10061108

[56] Keenan BP Van Loon K Khilnani AD Fidelman N Behr SC Atreya CE. Molecular and radiological features of microsatellite stable colorectal cancer cases with dramatic responses to immunotherapy. Anticancer Res. (2021) 41:2985–92. doi: 10.21873/anticanres.15080 PMC8631311 34083289

[57] Durando ML Menghani SV Baumann JL Robles DG Day TA Vaziri C. Four-year disease-free remission in a patient with POLE mutation–associated colorectal cancer treated using anti–PD-1 therapy. JNCCN J Natl Compr Cancer Netw. (2022) 20:218–23. doi: 10.6004/JNCCN.2021.7115 35276675

[58] Li S Zhang J Du W Ren X Zhang X. Pathologic complete response to immune checkpoint inhibitor in a stage IIIB ovarian clear cell carcinoma patient with POLE mutation resistant to platinum-based chemotherapy: a case report. Gland Surg. (2022) 11:1562–7. doi: 10.21037/gs-22-420 PMC9547720 36221285

[59] Tailored Adjuvant Therapy in POLE-mutated and p53-wildtype Early Stage Endometrial Cancer - Full Text View - ClinicalTrials.gov . Available online at: https://clinicaltrials.gov/study/NCT04705649 (Accessed March 13th, 2024)

[60] van den Heerik ASVM Horeweg N Nout RA Lutgens LCHW van der Steen-Banasik EM Westerveld GH. PORTEC-4a: international randomized trial of molecular profile-based adjuvant treatment for women with high-intermediate risk endometrial cancer. Int J Gynecol Cancer. (2020) 30:2002–7. doi: 10.1136/ijgc-2020-001929 PMC7788476 33046573

[61] Refining adjuvant treatment in endometrial cancer based on molecular features: The RAINBO clinical trial program. Int J Gynecol Cancer. (2022) 33:109–17. doi: 10.1136/ijgc-2022-004039 PMC9811074 36600534

